# Evaluation of a Community-based Quality Improvement Smoking Cessation Program Using the Ottawa Model During COVID-19

**DOI:** 10.1016/j.cjco.2026.01.003

**Published:** 2026-01-14

**Authors:** Salma Mahmoodianfard, Ayah Rashwan, Javad Heshmati, Terefe Gelibo, Mustafa Coja, David Sabapathy, Shamara Baidoobonso, Karen Phillips, Laura MacDonald, Laura Lee Noonan, Kerri-Anne Mullen, Hassan Mir

**Affiliations:** a University of Ottawa Heart Institute, Ottawa, Ontario, Canada; b Faculty of Medicine, University of Ottawa, Ottawa, Ontario, Canada; c Ottawa Model for Smoking Cessation, University of Ottawa Heart Institute, Ottawa, Ontario, Canada; d Chief Public Health Office, Charlottetown, Prince Edward Island, Canada

**Keywords:** heaviness of smoking, nicotine dependence, smoking cessation, tobacco abstinence

## Abstract

**Background:**

Tobacco smoking remains a major preventable cause of morbidity and mortality worldwide. In Canada, despite substantial declines in national smoking prevalence over the past 2 decades, important regional disparities persist, with some provinces reporting rates above the national average. This study assessed changes in smoking and cessation outcomes among participants in the Prince Edward Island (PEI) Smoking Cessation Program (SCP), adapted from the Ottawa Model for Smoking Cessation (OMSC) during the COVID-19 pandemic.

**Methods:**

This pre-post study evaluated the PEI SCP (2019–2021) among participants completing baseline and 6-month follow-up surveys. Primary outcomes were 7-day and 30-day point-prevalence abstinence at 6 months.

**Results:**

Of all tobacco users enrolled in the program (n=1,707), a total of 732 (42.9%) responded to both baseline and 6-month surveys. At the 6-month follow-up, 32.2% of participants achieved 7-day point-prevalence abstinence, and 27.5% of responders reported 30-day point-prevalence abstinence. Nicotine dependence level was significantly reduced (p < 0.001), with the proportion of participants in the low Heaviness of Smoking Index (HSI) category increasing from 17.0% at baseline to 48.7% at follow-up. While 66.5% felt that service quality was unaffected by COVID-19, 29.7% reported pandemic-related challenges to quitting, mainly stress (61.0%) and personal life challenges (31.9%).

**Conclusions:**

Findings from the PEI SCP showed that about one-third of respondents achieved smoking abstinence at 6 months, despite the COVID-19 pandemic. These results support continued implementation of evidence-based programs like OMSC in community health settings and highlight the program’s resilience under constrained healthcare conditions.

## Résumé

**Contexte:**

Le tabagisme reste une cause majeure évitable de morbidité et de mortalité au Canada, avec des différences régionales persistantes dans sa prévalence. Cette étude a évalué les changements dans les résultats en matière de tabagisme et de sevrage tabagique chez les participants au Programme d'abandon du tabac (PAT) de l'Île-du-Prince-Édouard (Î.-P.-É.), adapté du modèle d’Ottawa pour l'abandon du tabac (MOAT) pendant la pandémie de COVID-19.

**Méthodologie:**

Cette étude pré-post a évalué le PAT de l'Î.-P.-É. (2019-2021) auprès des participants ayant répondu aux questionnaires de référence et de suivi à 6 mois. Les critères principaux étaient la prévalence ponctuelle de l'abstinence à 7 jours et à 30 jours, mesurée au bilan des 6 mois.

**Résultats:**

Sur l'ensemble des fumeurs inscrits au programme (n = 1 707), 732 (42,9 %) ont répondu aux enquêtes initiales et à 6 mois. Au suivi à 6 mois, 32,2 % des participants ont atteint une abstinence ponctuelle de 7 jours, et 27,5 % des répondants ont déclaré une abstinence ponctuelle de 30 jours. Le niveau de dépendance à la nicotine a été réduit significativement (*p* < 0,001), la proportion de participants dans la catégorie « faible » de l'indice de tabagisme passant de 17,0 % au début de l'étude à 48,7 % lors du suivi. Alors que 66,5 % estimaient que la qualité du service n'avait pas été affectée par la COVID-19, 29,7 % ont signalé des difficultés liées à la pandémie pour arrêter de fumer, évoquant principalement le stress (61,0 %) et des difficultés personnelles (31,9 %).

**Conclusions:**

Les résultats du PAT de l'Î.-P.-É. ont montré qu'environ un tiers des répondants avaient réussi à arrêter de fumer au bout de 6 mois, malgré la pandémie de COVID-19. Ces résultats plaident en faveur de la poursuite de la mise en œuvre de programmes fondés sur des données probantes tels que le MOAT dans les établissements de santé communautaires et soulignent la résilience du programme dans des conditions de soins de santé limitées.

Tobacco use continues to be a leading cause of preventable illness and death, contributing to chronic diseases such as cardiovascular conditions, respiratory illnesses, and several types of cancer.^1^ In 2003, the **W**orld **H**ealth **O**rganisation (WHO) launched an international treaty called the **F**ramework **C**onvention on **T**obacco **C**ontrol (FCTC) to protect public health and reduce the economic burden caused by tobacco use.^2^ Despite the adoption of comprehensive tobacco control measures, smoking remains a major public health issue, with an estimated 1.25 billion adults worldwide who are currently smoking cigarettes. Of these, more than 60% (> 750 million people) are estimated to have a desire to quit. Nevertheless, approximately 70% of these users lack access to effective cessation services, primarily as a result of constrained resources within health care systems.^3^

In Canada, the federal government has set a goal to reduce tobacco consumption to < 5% of the population by the year 2035, to address the serious health impacts of tobacco use.^4^ Over the past 2 decades, smoking rates have consistently diminished, with the prevalence of smoking falling from approximately 25% in 1999 to 10.3% in 2020.^5^^,^^6^ Despite this national decline, certain populations, provinces, and territories continue to experience higher prevalence rates. For example, 13.4% of residents in the province of **P**rince **E**dward **I**sland (PEI) reported smoking daily in 2017-2018 compared with the national average of 10.9% during the same period. This underscores the importance of implementing tailored smoking cessation interventions that can effectively meet the distinct needs of diverse communities.

There is evidence that systematic and system-based approaches are effective in helping patients quit smoking, especially when pharmacotherapy is combined with counselling and postdischarge support.^7^, ^8^, ^9^, ^10^, ^11^ The **O**ttawa **M**odel for **S**moking **C**essation (OMSC) is among the best known structured, evidence-based approaches to addressing tobacco dependence within the health care settings.^8^^,^^11^^,^^12^ The program has been implemented in approximately 500 sites across Canada.^11^ It has also been implemented in 8 countries, including England, where the National Health Service has adopted and is implementing the OMSC as part of its long-term plan.^13^ The model consists of screening and documenting smoking status for all patients, providing brief counselling and pharmacotherapy, and offering follow-up support. In a multihospital evaluation conducted in eastern Ontario, implementation of the OMSC was associated with a substantial improvement in long-term quit rates, with a confirmed 6-month continuous abstinence rate of 29.4% compared with 18.3% prior to implementation.^14^ The OMSC has also been linked to significant reductions in hospital readmission rates, emergency department visits, and overall mortality rates up to 2 years postintervention, highlighting the model’s extensive impact on patient health and the health care system.^8^ Although originally developed for use in hospital settings, the OMSC has since been successfully adapted for primary care clinics and community settings,^15^^,^^16^ demonstrating its scalability across diverse health care environments.

In 2019, the PEI **S**moking **C**essation **P**rogram (SCP) was initiated, using the OMSC in a community setting to assist PEI residents in quitting smoking. It implemented evidence-based methods involving behavioural counselling, pharmacotherapy, and voluntary follow-up.^17^ In the current study we aimed to identify changes in smoking behaviour and cessation outcomes after implementation of the PEI SCP, using data collected between 2019 and 2021. The findings are intended to inform the continued development of provincial smoking cessation services and sustained implementation of the program in health care settings.

## Methods

### Study design

In this study we used a pre/post prospective observational design to describe changes in smoking behaviour and cessation outcomes among participants in the PEI SCP, which is informed and supported by the OMSC program. The sample includes program participants who completed baseline and 6-month follow-up evaluations.

### Program description

The PEI SCP is a publicly funded, universal cessation program that provides funding as a payer of last resort, covering costs when no other funding is available. It provides both counselling and pharmacotherapy, including nicotine replacement therapy (NRT) or prescription medication, to PEI residents who smoke or use other tobacco products.^17^ Participants receive a single continuous course (6-18 weeks) of NRT or prescription medication. The SCP was delivered by Health PEI through primary care networks (PCNs). Program development, data management, and 6-month follow-up were led by the **C**hief **P**ublic **H**ealth **O**ffice (CPHO), with support from the OMSC team.

PCNs are community-based clinics that are geographically distributed throughout PEI. These networks ensure residents of PEI can access primary care services close to home. Participants were enrolled across all 5 PCNs in PEI: Kings, Queens East, Queens West, East Prince, and West Prince.

Health care providers at PCNs across PEI were trained by OMSC staff to systematically identify all individuals who smoke, regardless of the reason they were accessing care (ask), provide brief counselling (advise and assess), and act (initiate medications and refer to PEI SCP) during primary care visits. Individuals who smoked and agreed to a referral were referred to PEI SCP for ongoing counselling, follow-up, and pharmacotherapy.

Registered nurses (RNs) serve as the first point of contact. At the intake appointment, RNs trained in the OMSC program reviewed each participant’s smoking history and readiness to quit and created a personalized quit plan. This included selecting pharmacotherapy options (NRT or prescribed medications), preparing for potential withdrawal symptoms, and offering follow-ups. Appointments were originally offered in person or in group sessions lasting 30-60 minutes. However, due to the COVID-19 pandemic, delivery shifted to telephone only. After the pandemic, program delivery transitioned to a hybrid model with both in-person and telephone appointments.

Participants picked up cessation products at local pharmacies. Prescriptions were required for medications such as varenicline or bupropion, which are commonly prescribed evidence-based smoking cessation aids.^18^ During the program, participants had access to optional follow-up counselling, delivered either in person or by telephone through PCN RNs. In addition to SCP counselling support, nurses could refer participants to supplementary services, such as the Smokers’ Helpline (phone and online services), Health PEI’s COPD Clinic, and mental health and addictions programs, depending on individual needs.

### Participant recruitment

**Figure 1:**
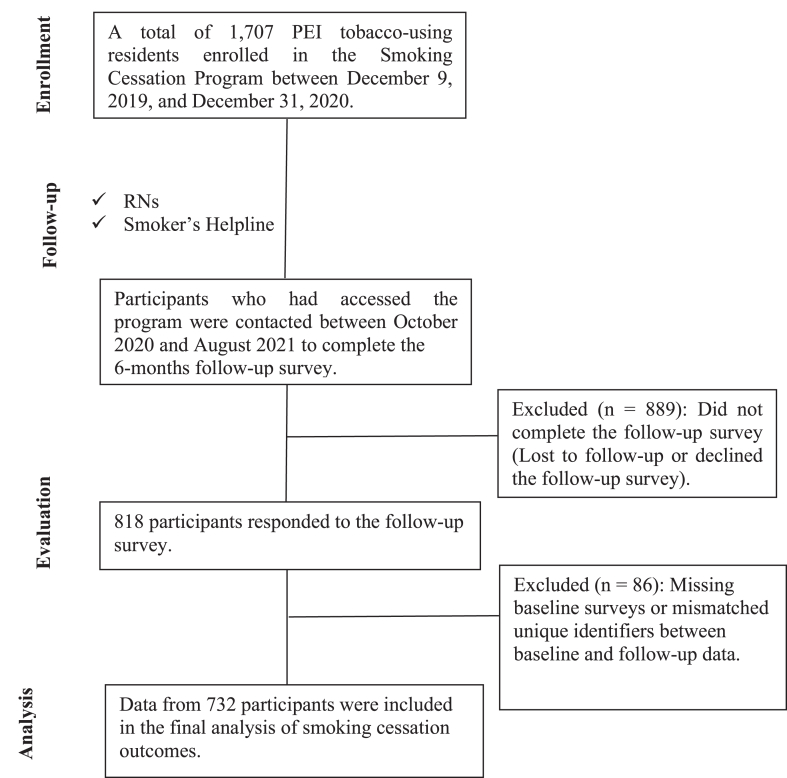
Flow of participants through the Prince Edward Island Smoking Cessation Program. RN, registered nurse.

Participants who accessed the SCP between December 9, 2019, and December 31, 2020, were contacted by telephone for the 6-month follow-up evaluation. To maximize response rates for the 6-month evaluation, multiple telephone call attempts (up to 6) were made when necessary to reach participants. The evaluation aimed to assess the program’s impact and gather participants’ experiences. The flow of participants through the study is illustrated in Figure 1. All participants provided written consent at program enrolment through a standardized consult form, which authorized Health PEI to use their personal health information for program evaluation and monitoring in accordance with the Health Information Act (R.S.P.E.I., 1988, Cap. H-1.41). Additional verbal consent was obtained at the beginning of telephone follow-up interviews. Participation was voluntary, with the right to withdraw at any time, and confidentiality was protected through coded identifiers and aggregate reporting.

### Measures

In this study we examined basic participant information such as demographics, patterns of smoking, and quitting success. Changes in cessation outcomes were assessed using self-reported 7-day and 30-day point prevalences of abstinence at 6-month follow-up, which were defined as the primary outcomes. For the 7-day point prevalence outcome, participants were asked, “At present, do you smoke daily, occasionally, or not at all?” Those who selected “not at all” were classified as abstinent. Interviewers included the time duration of the past week for clarity. If reporting “occasionally,” then they were asked to report the approximate number of cigarettes smoked over the past 7 days. For the 30-day point-prevalence outcome, participants were asked, “Have you smoked a cigarette or used other tobacco, even a puff, in the last 30 days?” Those who responded “no” were classified as abstinent. Individuals who quit on the day of the follow-up interview were classified as not abstinent. Other effectiveness outcomes included changes in nicotine dependence using the **H**eaviness of **S**moking **I**ndex (HSI), as well as smoking and vaping patterns over time. The HSI was calculated at baseline and at the 6-month follow-up for all participants based on the number of cigarettes smoked per day (0-3 points) and the time to first cigarette after waking (0-3 points).^19^ The score is used to categorize dependence level as low (0-1 point), medium (2-4 points), or high (5-6 points).^20^ In addition, the surveys assessed participants’ confidence in remaining smoke-free using a structured 5-point Likert-type scale ranging from low to high confidence. The survey also assessed experiences with pharmacotherapy, perceptions of the impact of COVID-19 on smoking cessation efforts, and overall satisfaction with the program. These perception-related items were also collected using the 5-point Likert-scale response options (from strongly disagree to strongly agree), as shown in Supplemental Figures S1 and S2.

### Statistical analysis

Data from baseline and the 6-month follow-up were merged with Microsoft Excel using unique patient identifiers. The merged data set was then imported into IBM SPSS version 29.0.1.0 software (IBM Corp, Armonk, NY, USA) for statistical analysis. Descriptive statistics were used to summarize participants’ characteristics, pharmacotherapy use, changes in behaviour, and smoking cessation outcomes. Categorical variables are presented as frequency and percentage and continuous variables are presented as mean and standard deviation. To assess changes in nicotine-dependence levels over time, a McNemar-Bowker test was conducted to compare the distribution of participants across the HSI categories (low, medium, high) at baseline and at the 6-month follow-up. *P* < 0.05 was considered statistically significant.

## Results

In total, 1707 participants were enrolled in the PEI SCP during the study period. Of these, 818 responded to 6-month evaluation surveys. After removing those with missing baseline surveys or mismatch in unique identifiers between baseline and follow-up, the final sample included and analyzed data from 732 responders (42.9% of the total enrolment) (Fig. 1). The distribution of participants by intake location was as follows: Kings PCN (16.0%), Queens East PCN (26.2%), Queens West PCN (28.7%), East Prince PCN (19.6%), and West Prince PCN (9.5%).

### Participants’ characteristics

Table 1 presents the demographic and smoking-related characteristics of the study population (n = 732). The mean age was 51.8 ± 15.3 years, sex was equally distributed (50.3% men, 49.7% women), and more than half (55%) of participants reported having a high school education or less. The mean age at which the responder’s first started smoking was 16.0 ± 4.3 years. At baseline, 99.2% of participants reported tobacco use in the previous 6 months, and 95% reported tobacco use in the 7 days before enrolment. On average, the participants smoked their first cigarette within 19.8 ± 22.6 minutes of waking, with a median of 10 minutes. Their average daily cigarette consumption was 21.5 ± 15.9, with a median of 20 cigarettes per day. Over two thirds (67.4%) of participants reported being regularly exposed to second-hand smoke.

**Table 1:** Basic characteristics of study patients

Characteristic	n (%) or %
Age, years	
< 18	9 (1.3%)
18-24	9 (1.3%)
24-45	195 (27.2%)
45-60	259 (6.2%)
> 60	244 (34.1%)
Sex	
Male	368 (50.3%)
Female	364 (49.7%)
Education level	
Less than high school	183 (25.3%)
High school diploma, certificate or equivalent	218 (30.1%)
Some postsecondary education without degree, certificate, or diploma	54 (7.5%)
Registered apprenticeship or trades certificate or diploma	33 (4.6%)
College, or other non-university certificate or diploma	176 (24.3%)
University degree (bachelor’s, master’s, or doctoral)	60 (8.3%)
Age at smoking initiation, years	
< 10	28 (3.9%)
10-18	560 (78.8%)
18-24	93 (13.1%)
24-30	21 (3.0%)
>30	9 (1.3%)
First cigarette after waking	
< 5 min	37.1%
5-30 min	51.1%
31-60 min	9.3%
> 60 min	2.5%
Cigarettes smoked per day	
1-9	45 (6.3%)
10-19	212 (29.8%)
20-29	350 (49.2%)
30-39	51 (7.2%)
> 40	53 (7.5%)
Exposure to second-hand smoke	456 (67.4%)

During the intake session, 67.3% of participants intended to use NRT while 31.7% opted to receive prescription medication, and a small proportion (≈ 1%) selected no pharmacotherapy. Among participants who completed the 6-month follow-up evaluation, medication dispensing data were available for all respondents. Of those who received an NRT recommendation at intake, 87.6% elected to use the nicotine patch, whereas 12.4% used a short-acting NRT product. Based on dispensing records at follow-up, 60.0% of participants received the nicotine patch, 8.4% received a short-acting NRT product, 27.5% received varenicline, and 4.1% received bupropion.

### Smoking cessation outcomes

The participants’ smoking behaviours and related factors, including their current smoking status, confidence in remaining smoke-free, and changes in smoking and vaping behaviour since enrolling in the program, are summarized in Table 2. At the 6-month follow-up, 32.2% of participants reported achieving 7-day point-prevalence abstinence. In addition, 30-day point-prevalence abstinence was reported at 27.5%. Among participants who achieved 7-day and 30-day point-prevalence abstinence, 91.8% and 92.9%, respectively, rated their confidence in remaining smoke-free as 4 or higher on the 5-point Likert scale. To assess whether cessation outcomes varied by pharmacotherapy type, we compared 7-day point-prevalence abstinence at 6 months across 2 treatment categories: NRT (patch or short-acting NRT) and prescription medications (varenicline or bupropion). Quit rates were comparable between groups (NRT: 31.9%; varenicline or bupropion: 32.9%), indicating no meaningful differences in cessation success based on pharmacotherapy used (Supplemental Table S1).

**Table 2:** Participant outcomes at 6-month follow-up

Variable	n (%)
Smoking status at the present (n = 732)	
Not at all	236 (32.2%)
Occasionally	67 (9.2%)
Daily	429 (58.6%)
Confidence in remaining smoke-free among those who quit (n = 233) (1-5 scale)	
1 (low)	1 (0.4%)
2	4 (1.7%)
3	14 (6.0%)
4	51 (21.9%)
5 (high)	163 (70.0%)
Smoking status in past 30 days	
Not smoked or used tobacco	201 (27.5%)
Smoked or used tobacco	527 (72.2%)
Do not know	2 (0.3%)

The HSI scores are presented in Table 3. At baseline, 83.0% of subjects were found to be in the medium to high nicotine-dependence range, whereas, at 6-month follow-up, this proportion decreased to 50.3%, representing a substantial reduction in nicotine-dependence levels over time. The observed changes in nicotine dependence were statistically significant (*P* < 0.001).

**Table 3:** Heaviness of smoking index scores at baseline and 6-month follow-up

HSI	Baseline, n (%)	Follow-up, n (%)	*P* value∗
Low (0-2)	119 (17%)	312 (48.7%)	< 0.001
Medium (3-4)	408 (58.2%)	235 (35.6%)	
High (5-6)	174 (24.8%)	94 (14.7%)	

HSI, Heaviness of Smoking Index.

∗ Statistical significance level set at *P* < 0.05.

### Changes in smoking and vaping behaviour

At follow-up, 68.3% of respondents reported smoking less or not at all compared with baseline. Only 4.3% reported smoking more compared with when they enrolled in the PEI smoking cessation program. In terms of vaping behaviour, 9.6% of respondents started vaping and 1.0% reported an increase in vaping since enrolling in the program (Table 4). Of the 70 participants who reported starting vaping since enrolling, 40.0% had quit smoking, 11.4% smoked occasionally, and 48.6% continued to smoke daily.

**Table 4:** Smoking and vaping behaviour changes postenrolment

Change in smoking behaviour since enrolment in program	n (%)
Not smoking	227 (31.3%)
Smoking less	268 (37.0%)
Smoking the same	199 (27.4%)
Smoking more	31 (4.3%)
Vaping behaviour since enrolment in program (yes or no)	
Not vaping	653 (89.4%)
Started vaping	70 (9.6%)
Increased vaping	7 (1.0%)
Smoking status among those who started vaping (n = 70)	
Not smoking	28 (40.0%)
Smoking occasionally	8 (11.4%)
Smoking daily	34 (48.6%)

### Perceived impact of the program on smoking cessation efforts

Participants’ perceptions of the PEI SCP’s impact on their smoking cessation efforts are shown in Supplemental Figure S1. Most participants (71%) agreed or strongly agreed that the program was helpful in supporting their efforts to quit or reduce smoking.

### Impact of COVID-19 on smoking cessation

A total of 66.5% of participants disagreed or strongly disagreed that COVID-19 negatively impacted the quality of service they received, suggesting that most participants did not perceive a decline in service quality during the pandemic (Supplemental Fig. S2).

Notably, nearly 30% of participants reported that COVID-19 had a negative effect on their efforts to quit smoking. Among these participants, the primary contributing factors were COVID-19–related stress, reported by > 61% either alone or in combination with other challenges, and personal life circumstances, reported by 31.9%, which interfered with continued engagement in the program. The participants’ reported COVID-19–related challenges to cessation efforts are summarized in Supplemental Figure S3.

## Discussion

This study was a pre/post evaluation of the PEI SCP with participants who completed the 6-month follow-up evaluation. At 6-month follow-up, the program showed promising quit rates, a significant reduction in nicotine dependence, and positive perceptions of service quality across the province despite the challenges of the COVID-19 pandemic.

Our findings suggest that residents of PEI who participated in this community-based program experienced meaningful improvements in cessation-related outcomes. The program was effective in supporting many participants in their quit attempts. At 6-month follow-up, 32.2% of participants reported 7-day point-prevalence abstinence and 27.5% reported 30-day point-prevalence abstinence. In addition, over two thirds (68.3%) reported either reducing or quitting. Among those who achieved abstinence, > 90% expressed a high level of confidence in remaining smoke-free at the time of follow-up. Furthermore, there was a significant reduction in nicotine dependence, with the proportion of participants classified as having medium to high dependence (based on HSI score) decreasing from 83.0% at baseline to 50.3% at 6-month follow-up. The overall use of cessation aids at follow-up, particularly the nicotine patch (60.0%) and varenicline (27.5%), reflects active engagement with recommended treatments for quitting smoking. This aligns with clinical guidelines emphasizing the importance of using pharmacotherapy to increase the likelihood of successful smoking cessation efforts.^11^^,^^21^

The PEI SCP was adapted from and supported by the OMSC program and team. The OMSC combines best available evidence-based tools and protocols with principles of implementation science and change management to enhance community-based cessation services, with a focus on patient care.^14^ The program has been confirmed to significantly improve rates of smoking cessation in diverse locations, including the hospital (Ontario and England)^22^^,^^23^ and outpatient (diabetes clinic, oncology clinics, lung cancer screening) settings.^24^, ^25^, ^26^ Previous evaluations of OMSC implementation have also demonstrated its broad real-world effectiveness, showing significant improvements in rates of smoking cessation, clinical outcomes including mortality, and health care utilization (30 days, 1 year, and 2 years).^8^^,^^27^ These improvements also led to a significant reduction in health care costs by ∼ 20%, confirming it as a cost-effective intervention. This PEI SCP implementation further demonstrates OMSC effectiveness in a province-wide community setting.

The shift in HSI categories in this study suggests a positive impact of the PEI SCP on participants’ smoking behaviours and underscores its role in achieving tobacco-related harm reduction,^28^ even among individuals who did not achieve full abstinence. Withdrawal symptoms have been identified as a key barrier to successful cessation.^29^ Previous research has shown that, although individuals with higher nicotine dependence are more likely to attempt quitting,^29^ they are generally less successful in maintaining long-term abstinence.^30^ On the other hand, lower nicotine dependence and higher self-efficacy are associated with greater success in future quit attempts^31^ and a reduced risk of relapse.^32^^,^^33^ Therefore, shifting the HSI curve toward lower dependence levels increases the likelihood of future successful quit attempts. Another commonly cited barrier identified by individuals trying to quit smoking is the cost of cessation pharmacotherapies.^34^^,^^35^ In this context, the PEI SCP’s provision of full coverage of one course of pharmacotherapy, which allowed participants to choose between NRT or prescription medications, reduced financial barriers and made options like varenicline more accessible. Nonetheless, there is strong evidence to support that combination NRT (long-acting patch with short-acting gum, lozenge, inhaler, or spray) is superior to the solo form of NRT.^36^ Future advocacy could focus on funding the provision of free combination NRT for all residents.

In addition to pharmacotherapy use, approximately 9.6% of participants reported initiating vaping after enrolment in the program, and 40.0% of these individuals had quit smoking. Vaping has emerged as a harm-reduction tool for smoking cessation among individuals seeking alternatives to combustible tobacco products.^11^^,^^37^^,^^38^ These results suggest that some people who smoke within the PEI SCP may use e-cigarettes to quit. Although this substitution may be acceptable within cessation programs, dual use should be discouraged. The long-term impacts of vaping on health outcomes are unclear and thus shared decision-making is vital to provide personalized care.^39^

The impact of the COVID-19 pandemic was multifaceted. Although 66.5% of participants reported no negative effect on service quality, nearly one third indicated that the pandemic had hindered their cessation efforts. Stress-induced relapses emerged as the most frequently reported barrier to quitting smoking during the pandemic. These findings are consistent with existing evidence showing increased tobacco use and relapse during the pandemic, particularly driven by elevated stress levels, feelings of loneliness, social isolation, boredom, and changes in routines.^40^, ^41^, ^42^ Nevertheless, despite these barriers, most participants were able to access the required support and medications throughout the public health crisis, highlighting the success of the PEI SCP in their ability to deliver high-quality care.

There are several limitations to this study. First, this was an observational study, limiting the ability to infer causality. Second, the study did not include a control group. However, the quit rate in this evaluation was > 30%, which is significantly higher than would be expected in a control group based on anecdotal experience in PEI and based on OMSC implementation in similar settings across Ontario. A cluster randomized controlled trial of OMSC implementation in 8 primary care clinics in Ontario with close to 500 participants showed a control group quit rate of 11% at 6-month follow-up.^43^ This occurred despite using a control group that would be considered stronger than usual care in the current evaluation. Moreover, a 2021 Cochrane Review of smoking cessation interventions in primary care (81 RCTs with > 112,000 participants) showed quit rates of 3%-4% among those receiving standard care.^44^ Third, the pre/post study design may introduce sources of bias, confounding, and cointervention. Unmeasured confounding variables, such as mental health status, concurrent substance use, or changes in social support, could have also influenced cessation outcomes, but they were not evaluated. The COVID-19 pandemic may have also confounded both smoking behaviours (eg, stress-related changes in habits) and program delivery (eg, shifts to virtual counselling). Nonetheless, this design was influenced by the COVID-19 pandemic with focus on rapid and feasible program evaluation and improvement. It is reassuring that the results align with previous studies leveraging more robust study design, despite the effect of the COVID-19 pandemic. Future evaluation of the PEI SCP would also confirm the positive findings noted in this evaluation. Fourth, participants included in our evaluation represent individuals who smoke, are interested in quitting, and willing to accept a referral to a smoking cessation program. This could have introduced referral or selection bias and thus the findings may not be generalizable to those who smoke and are not interested in quitting or being referred to a smoking cessation program. In addition, approximately 50% completed 6-month follow-up surveys, which may have also introduced selection bias and result in overestimation of program effectiveness. Nonetheless, loss to follow-up of this magnitude is common in smoking cessation program evaluations and may have been influenced by disruptions related to the COVID-19 pandemic.^45^ It is also reassuring that the results of this evaluation align well with previously published OMSC implementations, including studies that reported substantially higher 6-month follow-up response rates.^14^^,^^15^ Fifth, abstinence outcomes were self-reported rather than the “gold-standard” biochemical verification,^46^ which could introduce social desirability bias thus overestimating quit rates. Although this was planned to maximize feasibility, the COVID-19 pandemic would have made it challenging to perform in-person carbon monoxide testing for biochemical verification. Future evaluations could strengthen the reliability of cessation outcomes by measuring and adjusting for known confounders, incorporating biochemical verification methods, and repeating program evaluation to reproduce the findings of this study.

Despite the challenges posed by the COVID-19 pandemic, affecting patients, providers, and health care systems, this evaluation of the PEI SCP demonstrates high levels of satisfaction and effectiveness among participants. These findings are consistent with those of previous studies evaluating OMSC and underscore the program’s resilience and adaptability, even under constrained health care conditions.

## Conclusions

This study has demonstrated high satisfaction and effectiveness of OMSC implementation in PEI, with substantial reduction in nicotine dependence and high rates of smoking reduction or cessation. The program continued to provide high-quality support and effectively assisted participants in their efforts to quit smoking despite the challenges posed by the COVID-19 pandemic. Our findings highlight the importance of integrated behavioural counselling, pharmacotherapy, and follow-up in future smoking cessation interventions.

## References

[1] World Health Organization (2023). Tobacco.

[2] World Health Organization (2003). WHO framework convention on tobacco control. https://iris.who.int/bitstream/handle/10665/42811/9241591013.pdf.

[3] World Health Organization (2024).

[4] Health Canada (2023). Overview of Canada's Tobacco Strategy. https://www.canada.ca/en/health-canada/services/publications/healthy-living/canada-tobacco-strategy/overview-canada-tobacco-strategy.html.

[5] Reid J.L., Hammond D., Burkhalter R., Rynard V.L. (2022). Tobacco use in Canada: patterns and trends. 2022.

[6] Hersi M., Beck A., Hamel C. (2024). Effectiveness of smoking cessation interventions among adults: an overview of systematic reviews. Syst Rev.

[7] Rigotti N.A., Clair C., Munafo M.R., Stead L.F. (2012). Interventions for smoking cessation in hospitalised patients. Cochrane Database Syst Rev.

[8] Mullen K.A., Manuel D.G., Hawken S.J. (2017). Effectiveness of a hospital-initiated smoking cessation programme: 2-year health and healthcare outcomes. Tob Control.

[9] Streck J.M., Rigotti N.A., Livingstone-Banks J. (2024). Interventions for smoking cessation in hospitalised patients. Cochrane Database Syst Rev.

[10] Amaral L.M., Macêdo Â.C., Lanzieri I.O. (2020). Promoting cessation in hospitalized smoking patients: a systematic review. Rev Assoc Med Bras.

[11] Mir H., Eisenberg M.J., Benowitz N.L. (2025). Canadian Cardiovascular Society clinical practice update on contemporary approaches to smoking cessation. Can J Cardiol.

[12] Reid R.D., Pipe A.L., Quinlan B. (2006). Promoting smoking cessation during hospitalization for coronary artery disease. Can J Cardiol.

[13] National Health Service (2019). Smoking. https://www.longtermplan.nhs.uk/online-version/chapter-2-more-nhs-action-on-prevention-and-health-inequalities/smoking.

[14] Reid R.D., Mullen K.A., Slovinec D., Angelo M.E. (2010). Smoking cessation for hospitalized smokers: an evaluation of the Ottawa Model. Nicotine Tob Res.

[15] Papadakis S., Cole A.G., Reid R.D. (2016). Increasing rates of tobacco treatment delivery in primary care practice: evaluation of the Ottawa Model for Smoking Cessation. Ann Fam Med.

[16] Pipe A.L., Evans W., Papadakis S. (2022). Smoking cessation: health system challenges and opportunities. Tob Control.

[17] Chief Public Health Office, Government of Prince Edward Island (2022). Prince Edward Island Smoking Cessation Program evaluation report. https://www.princeedwardisland.ca/sites/default/files/publications/pei_smoking_cessation_report_2022_final.pdf.

[18] Zawertailo L., Mansoursadeghi-Gilan T., Zhang H. (2018). Varenicline and bupropion for long-term smoking cessation (the MATCH Study): protocol for a real-world, pragmatic, randomized controlled trial. JMIR Res Protoc.

[19] Heatherton T.F., Kozlowski L.T., Frecker R.C., Rickert W., Robinson J. (1989). Measuring the heaviness of smoking: using self-reported time to the first cigarette of the day and number of cigarettes smoked per day. Br J Addict.

[20] Hwang J.S., Lee C.M., Lee K., Kim C.Y. (2021). Nicotine dependence evaluated by urinary cotinine and Heaviness of Smoking Index among smokers, vapers, and dual users: a cross-sectional study using the Korea National Health and Nutrition Examination Survey data. Korean J Fam Med.

[21] Tobacco Use and Dependence Guideline Panel (2008). Treating Tobacco Use and Dependence: 2008 Update. https://www.ncbi.nlm.nih.gov/books/NBK63952/.D.G.

[22] Mullen K.A., Walker K.L., Hobler L.A. (2021). Performance obligations to improve delivery of hospital-initiated smoking cessation interventions: a before-and-after evaluation. Nicotine Tob Res.

[23] Evison M., Pearse C., Howle F. (2020). Feasibility, uptake and impact of a hospital-wide tobacco addiction treatment pathway: results from the CURE project pilot. Clin Med (Lond).

[24] Reid R.D., Malcolm J., Wooding E. (2018). Prospective, cluster-randomized trial to implement the Ottawa Model for Smoking Cessation in diabetes education programs in Ontario, Canada. Diabetes Care.

[25] Mullen K.A., Hurley K., Hewitson S. (2024). Cost-effectiveness of point of care smoking cessation interventions in oncology clinics. Br J Cancer.

[26] Evans W.K., Tammemägi M.C., Walker M.J. (2023). Integrating smoking cessation into low-dose computed tomography lung cancer screening: results of the Ontario, Canada pilot. J Thorac Oncol.

[27] Mullen K.A., Coyle D., Manuel D. (2015). Economic evaluation of a hospital-initiated intervention for smokers with chronic disease, in Ontario, Canada. Tob Control.

[28] Hatsukami D.K., Carroll D.M. (2020). Tobacco harm reduction: past history, current controversies and a proposed approach for the future. Prev Med.

[29] John U., Meyer C., Hapke U., Rumpf H.J., Schumann A. (2004). Nicotine dependence, quit attempts, and quitting among smokers in a regional population sample from a country with a high prevalence of tobacco smoking. Prev Med.

[30] Breslau N., Johnson E.O. (2000). Predicting smoking cessation and major depression in nicotine-dependent smokers. Am J Public Health.

[31] Lindberg A., Niska B., Stridsman C. (2015). Low nicotine dependence and high self-efficacy can predict smoking cessation independent of the presence of chronic obstructive pulmonary disease: a three year follow up of a population-based study. Tob Induc Dis.

[32] Lee S.E., Kim C.W., Im H.B., Jang M. (2021). Patterns and predictors of smoking relapse among inpatient smoking intervention participants: a 1-year follow-up study in Korea. Epidemiol Health.

[33] Herd N., Borland R., Hyland A. (2009). Predictors of smoking relapse by duration of abstinence: findings from the International Tobacco Control (ITC) Four Country Survey. Addiction.

[34] van den Brand F.A., Nagelhout G.E., Reda A.A. (2017). Healthcare financing systems for increasing the use of tobacco dependence treatment. Cochrane Database Syst Rev.

[35] Cahill K., Hartmann-Boyce J., Perera R. (2015). Incentives for smoking cessation. Cochrane Database Syst Rev.

[36] Lindson N., Chepkin S.C., Ye W. (2019). Different doses, durations and modes of delivery of nicotine replacement therapy for smoking cessation. Cochrane Database Syst Rev.

[37] Hartmann-Boyce J., Lindson N., Butler A.R. (2022). Electronic cigarettes for smoking cessation. Cochrane Database Syst Rev.

[38] Heshmati J., Pandey A., Benjamen J. (2025). Vaping cessation interventions: a systematic review and meta-analysis. Tob Control.

[39] Pipe A.L., Mir H. (2022). E-cigarettes reexamined: product toxicity. Can J Cardiol.

[40] Vanderbruggen N., Matthys F., Van Laere S. (2020). Self-reported alcohol, tobacco, and cannabis use during COVID-19 lockdown measures: results from a web-based survey. Eur Addict Res.

[41] Gendall P., Hoek J., Stanley J., Jenkins M., Every-Palmer S. (2021). Changes in tobacco use during the 2020 COVID-19 lockdown in New Zealand. Nicotine Tob Res.

[42] Yingst J.M., Krebs N.M., Bordner C.R. (2021). Tobacco use changes and perceived health risks among current tobacco users during the COVID-19 pandemic. Int J Environ Res Public Health.

[43] Papadakis S., McDonald P.W., Pipe A.L. (2013). Effectiveness of telephone-based follow-up support delivered in combination with a multicomponent smoking cessation intervention in family practice: a cluster randomized trial. Prev Med.

[44] Lindson N., Pritchard G., Hong B. (2021). Strategies to improve smoking cessation rates in primary care. Cochrane Database Syst Rev.

[45] Krieger N., LeBlanc M., Waterman P.D. (2023). Decreasing survey response rates in the time of COVID-19: implications for analyses of population health and health inequities. Am J Public Health.

[46] Sandberg A., Sköld C.M., Grunewald J., Eklund A., Wheelock Å.M. (2011). Assessing recent smoking status by measuring exhaled carbon monoxide levels. PLoS One.

