# Supplementary material for: Evaluation of a Community-based Quality Improvement Smoking Cessation Program Using the Ottawa Model During COVID-19
Source: CJC Open. 2026 Jan 14;8(5):607–15. doi: 10.1016/j.cjco.2026.01.003 (PMC13174204; doi:10.1016/j.cjco.2026.01.003)
Supplement: Supplementary Figures and Table [file mmc1.docx]

**Supplementary Materials**

**Supplemental Table S1: Six-month smoking outcomes by pharmacotherapy type**

| **Pharmacotherapy Group** | **Not smoking at 6 months (n)** | **Smoking at 6 months (n)** | **Total (n)** | **Quit Rate (%)** |
| --- | --- | --- | --- | --- |
| **NRT (patch or short-acting NRT)** | 160 | 341 | 501 | **31.9%** |
| **Prescription medication (varenicline or bupropion)** | 76 | 155 | 231 | **32.9%** |
| **Total** | 236 | 496 | 732 |  |


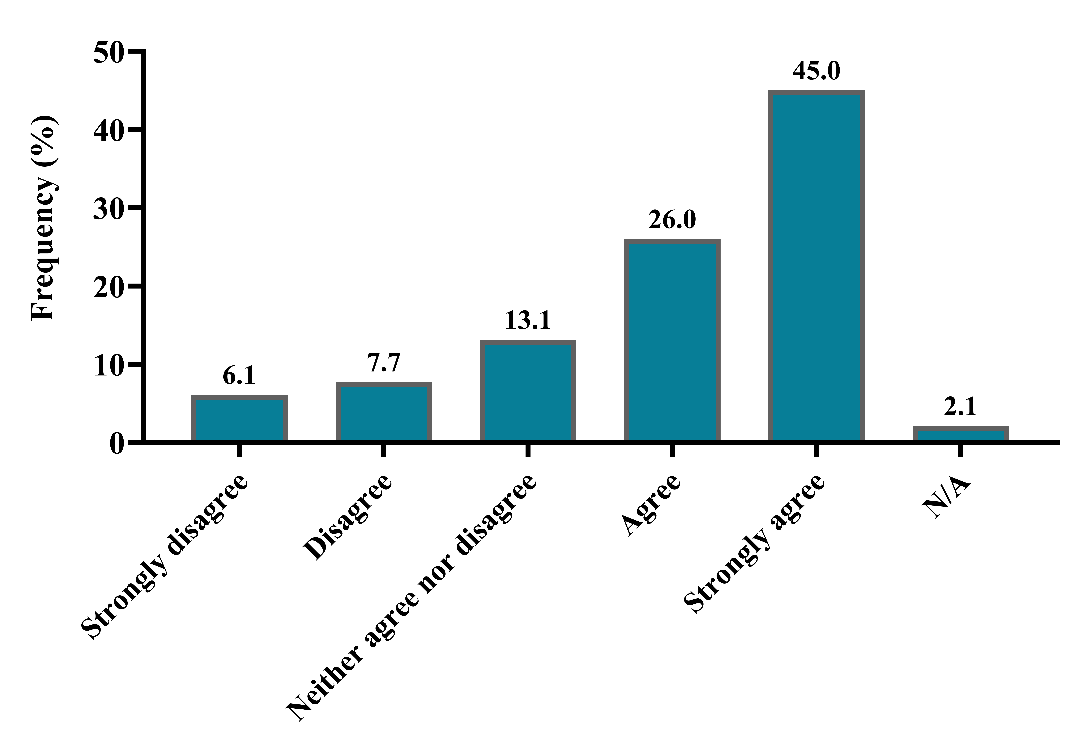


**Supplemental Figure S1: Participants’ Perceptions of the Impact of the PEI Smoking Cessation Program on Their Smoking Cessation Efforts**


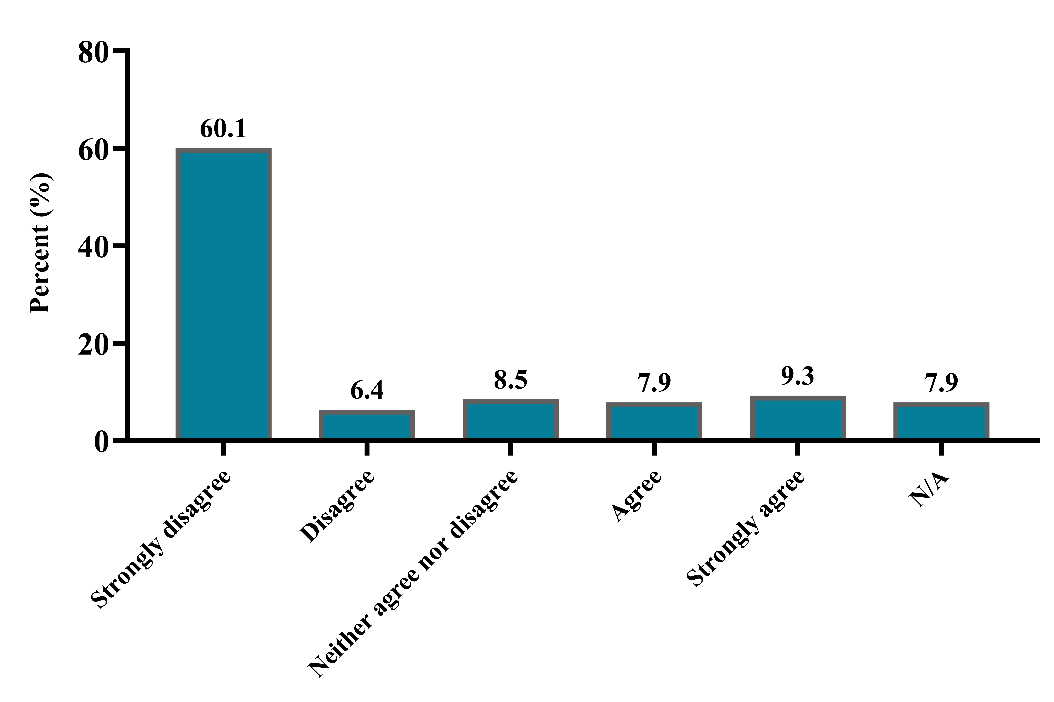


**Supplemental Figure S2. Participants’ perception on the Negative Impact of COVID-19 on the Quality of Service received During the Program.**


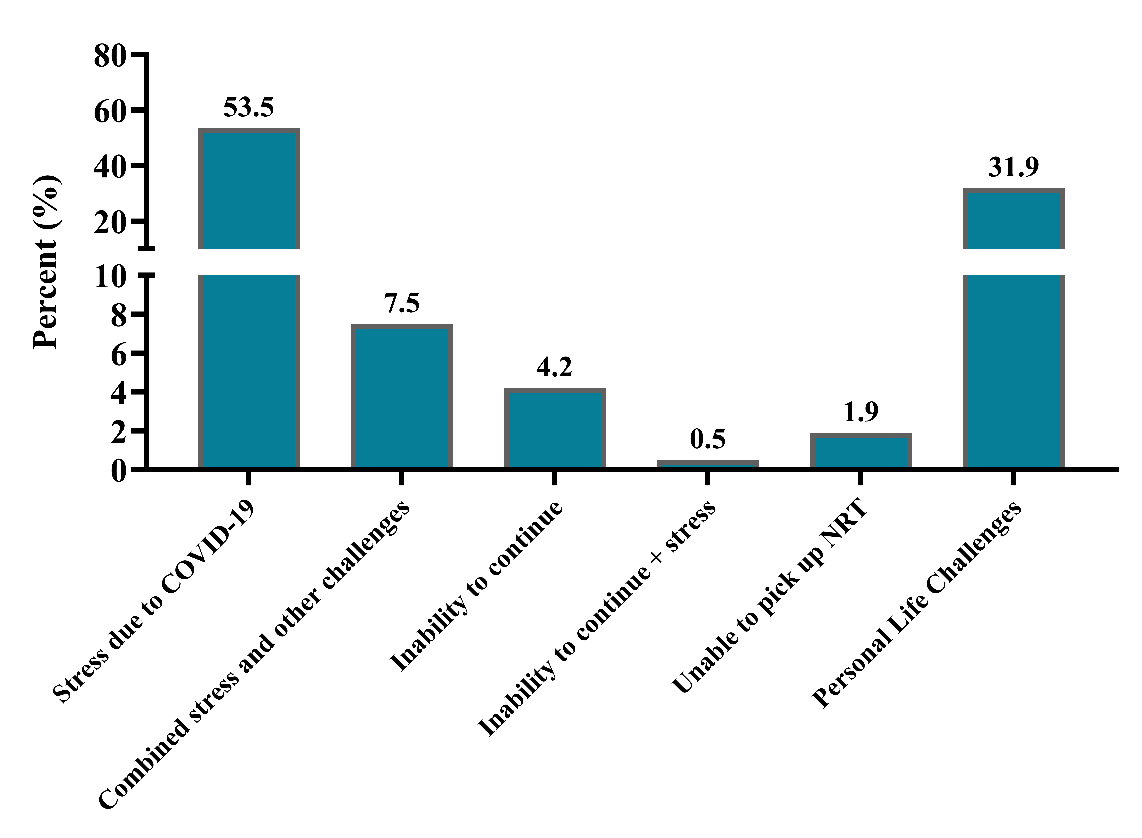


**Supplemental Figure S3: COVID-19–Related Barriers to Smoking Cessation**
